# Characteristics of deformation and damage of surrounding rock along the top roadway in the working face of an isolated island and its evolution law

**DOI:** 10.1038/s41598-024-63246-x

**Published:** 2024-07-02

**Authors:** Qing-Long Yun, Xiao-He Wang, Wu Jing, Wen-Bo Zhang, Xiao-Xiang Wei, Jiang-Hao Wang

**Affiliations:** School of Energy and Mining Engineering, China University of Mining and Technology (Bei Jing), Beijing, 100083 China

**Keywords:** Isolated island working face, Roadway along roof, Numerical simulation, Asymmetric expansion, Field measurement, Mineralogy, Coal

## Abstract

This study investigates the deformation and damage characteristics of the surrounding rock along the top return mining roadway of an isolated island working face at different stages and reveals its damage mechanism and evolution law. Utilizing a mine in Yangquan City, Shanxi Province, China, as the engineering background, this research employs FLAC 3D numerical simulation and on-site measurements. The findings suggest that the evolution of the plastic zone along the top roadway of the 15,106 island face is largely similar during both the excavation and mining periods. The plastic zones on either side of the roadway are expanding asymmetrically and gradually merging into the plastic zone of the coal pillar. In the destructive stage, the sub-gangs of the roadway are penetrated, indicating the progression into the plastic zone. The investigation points to extensive damage on the larger side of the roadway, the development of fissures, and the significant depth of damage as primary causes of roadway deformation. Moreover, the extent of the plastic zones on both sides of the roadway correlates positively with their relative distance. Continuous monitoring reveals an ongoing increase in roadway displacement, consistent with general observations in coal mining. The results provide valuable insights for optimizing support structures in similar mining environments.

## Introduction

As coal is a primary energy source in China, its role in the country's development is crucial^1^. Yangquan City in Shanxi Province hosts significant coal deposits. Nevertheless, the mining sequence design often results in isolated island working faces within the coal seam, which experience a mining influence distance three to five times greater than typical working faces. This increases the severity of mine pressure on the mining roadways^2–4^. The redistribution of surrounding rock stress due to mining disturbances is a key factor contributing to plastic failure zones in roadways^5,6^. Such disturbances can also trigger dynamic disasters such as rib spalling, roof falls, and even rock bursts^7–10^. Current research into the failure characteristics and spatial extent of surrounding rock in mining roadways includes theories such as the natural caving arch^11^, maximum horizontal stress theory, and axial strain theory^12,13^, supported by both theoretical analyses and field engineering practices. The radius of the plastic zone around a circular hole under hydrostatic pressure is calculated using the Fenner formula and Castenai formula^14–16^, furthering the understanding of deformation characteristics and failure morphology development in roadways. Ma Nianjie and others have developed the butterfly failure theory of roadway surrounding rock based on the plane strain model in elastic–plastic mechanics^17–22^. This theory posits that the plastic zone around a circular roadway with homogeneous rock typically manifests in circular, elliptical, or butterfly shapes under varying confining pressures, with derived criteria for different formations.

Numerous scholars have explored the failure mechanism characteristics and spatial extent of surrounding rock in mining roadways under various conditions^23–26^. Wu Xiangye et al.^27^ identified the mechanical mechanism behind the asymmetric expansion of the plastic zone by examining the failure mechanism of mining roadways on a gentle slope and at close proximity. They determined that the deviatoric stress defines the range of failure, while the principal stress dictates the direction of failure. The interaction between these factors leads to the asymmetric expansion of the plastic zone. Li Guanjun et al.^28^ investigated the failure mechanism of surrounding rock in gob-side entry driving under unstable overburden conditions, finding that roof cutting and pressure relief techniques can mitigate damage under dynamic loads. Wu Xingyu et al.^29^ examined the deformation and failure characteristics of surrounding rock in deep roadways under static and dynamic coupling stresses through numerical simulations. Their research suggests that horizontal stress has a more significant impact on roadway deformation than vertical stress, providing a foundation for support design and optimization under specific geological and geotechnical conditions. Yang Yu et al.^30^ developed a mechanical calculation model for composite roof roadways based on the actual characteristics of coal seams and the slate of roof and floor in a Guizhou coal mine, deriving expressions for the separation and instability limit load of the composite roof. Zhang Meichang et al.^31,32^ explored rockburst hazard prediction and forecasting using particle swarm algorithms and neural networks. They proposed an impact risk assessment method based on BP neural networks and modified the kinematic relationship by accounting for thickness stretching in the theory of coupled stress to study the kinetic equations of sandwiched hollow microspheres. Through theoretical analysis, numerical simulations, and field experiments, Sun Xiaoming et al.^33–35^ proposed a method that combines high prestress constant resistance supports with roof-cutting technology to effectively improve the stability of roadways in extra-thick coal seams. This approach examines the deformation and failure mechanisms of mining roadways and the combined support system under conditions of significant burial depth. Wang Min et al.^36–42^ conducted extensive research on the mechanical properties of various rock types using the Schmidt hammer test , multiple algorithms and prediction models. They proposed several empirical formulas and analyzed the rock damage process from different perspectives, providing guidance for related engineering practices.

The stress on the surrounding rock of the isolated island working face is redistributed following multiple mining operations. During the process of roadway excavation and working face mining, both the peak value and the influence range of the support pressure increase, leading to varying degrees of damage to the roadway. This damage severely affects both the safety and efficient production of the coal mine. In this study, using numerical simulations and field measurements, we analyze the deformation and failure laws of the surrounding rock of the roadway along the roof of the island working face. We detail the evolution process, providing a theoretical basis for the maintenance and stability control of the coal mine roadway, thereby safeguarding the mine's safe production.

## Methods

### Engineering background

**Figure 1:**
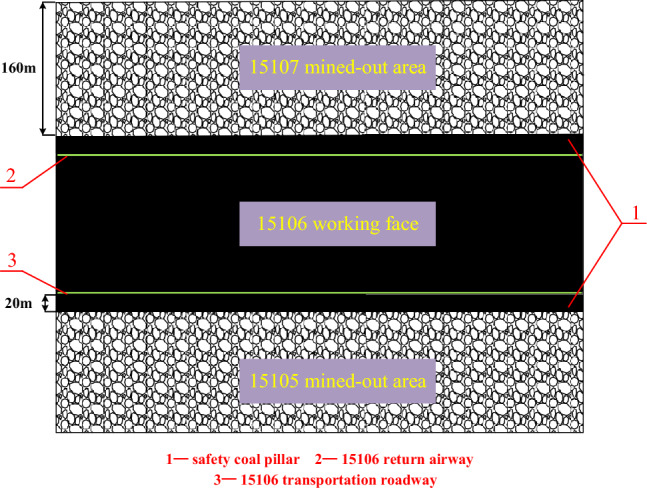
Relative position of working face and mined-out area.

A coal mine located in Yangquan City, Shanxi Province, China, predominantly mines the No.15 coal seam. This seam has an average thickness of 5.5 m and is buried approximately 370 m below the surface, characterized as a near-horizontal structure. The direct roof over the coal seam primarily consists of sandy mudstone and mudstone, while the main roof is composed of dense, fine-grained sandstone. The floor of the coal seam comprises fine-grained sandstone and mudstone. The working face of the No.15 coal seam spans 160 m. The mining roadway runs along the roof of the coal seam. Both the transportation roadway and the return air roadway measure 5 m in width and 3.4 m in height. The 15,106 working face of the mine is situated in the western part of the underground mining area. It is an isolated working face, flanked by the 15,107 working face to the west and the 15,105 working face to the east. Mining operations at these adjacent faces have been completed and sealed. A safety coal pillar of 20 m has been maintained. The relative position of these elements is illustrated in Fig. 1.

**Figure 2:**
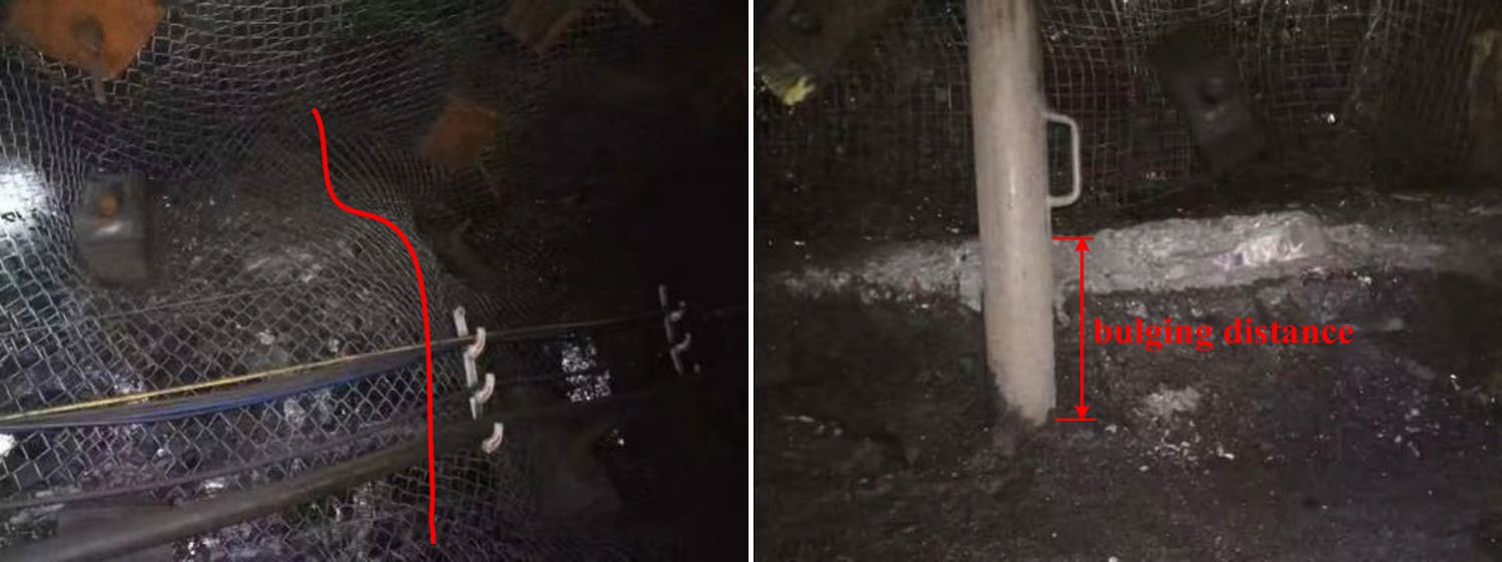
The side and bottom of the roadway are seriously bulging.

According to future plans for the mine, both the transportation and return air roadways of the 15,106 working face are scheduled for excavation, followed by the mining of this face. The mining roadway is excavated along the roof of the coal seam. During the excavation phase, the roadway experienced side heave and floor heave disasters, resulting in poor conditions on the sides and floor of the roadway. Throughout the mining period of the 15,106 working face, repeated mining activity led to significant convergence on both sides of the mining roadway, with serious deformation and damage, particularly in the middle and lower sections. Additionally, a noticeable floor heave phenomenon was observed, which severely impacted the mine's safe production and transportation operations. Daily cleanup of the bulging sections of the roadway floor was necessary. The relative displacement between the roof and floor was significant, as shown in Fig. 2, although the roof remained relatively intact. In order to analyze the deformation and failure characteristics of the surrounding rock of the mining roadway at the 15,106 working face, a combination of numerical simulation and field observation was employed to investigate and elucidate its spatiotemporal evolution.

### Site measuring points arrangement

The measurement of surface displacement is crucial for monitoring the relative movement of the roadway sides at various positions as the working face progresses, and for understanding the time-dependent displacement patterns of the roadway surface. A mine laser range finder is employed to measure the changes in spacing between the two sides of the roadway. Additionally, to directly observe the development and damage of cracks in the surrounding rock, a borehole peeper is used to position the measuring points at the site.

**Figure 3:**
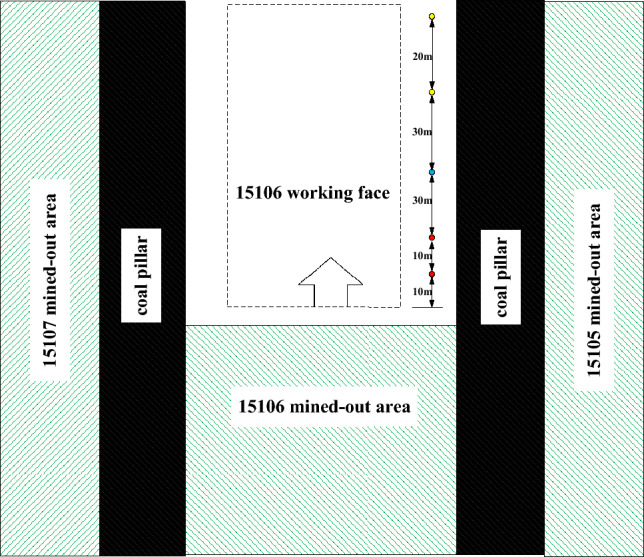
Measurement point layout.

In light of the actual production conditions at the 15,106 working face in the transportation roadway, measuring points for surface displacement are set up starting 80 m from the working face, with subsequent points every 10 m, totaling three points. Simultaneously, borehole peeping points are positioned at 50 m, 80 m, and 100 m from the 15,106 working face. These boreholes are located in the lower part of the roadway and have a depth of 6 m. There are three borehole peeping points and three surface displacement measuring points. The layout of these points is depicted in Fig. 3. In this figure, borehole peeping points are marked in red, surface displacement points in yellow, and two other points are shown in blue.

### Numerical simulation scheme

Following the mining operations at the coal face, the original stress balance in the surrounding rock of the roadway is disrupted, leading to stress redistribution and the emergence of bearing pressure, which intensifies stress concentration around the roadway. The surrounding rock may experience varying degrees of disturbance due to mining activities. When the stress exceeds the yield limit, plastic failure can occur, potentially leading to disasters such as bulging of the surrounding rock. The damage to the surrounding rock in the mining roadway of the isolated working face is expected to be more severe, with correspondingly intense mining pressure.

**Figure 4:**
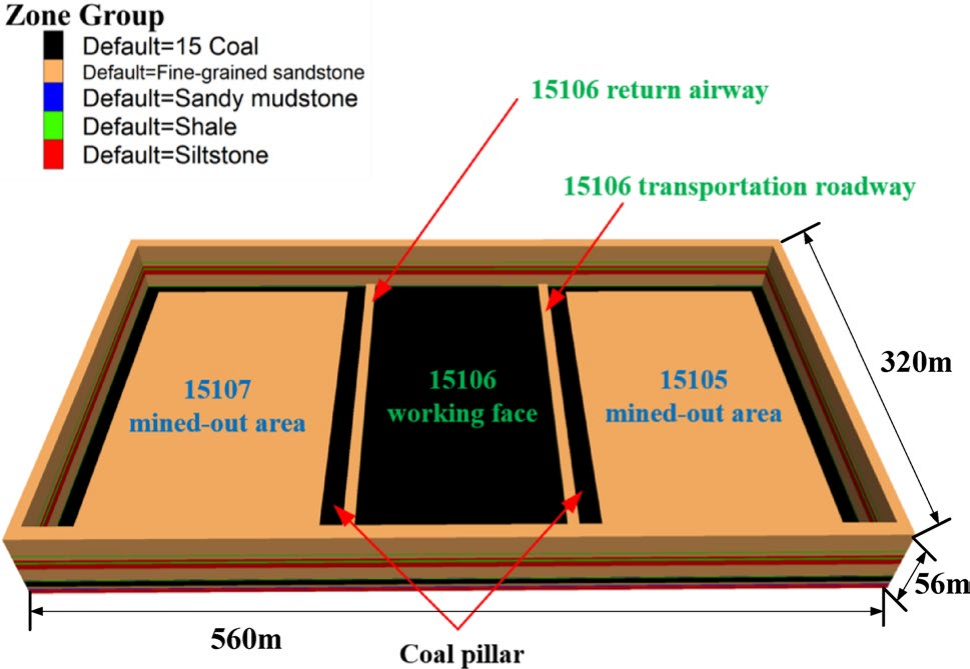
Numerical model diagram.

In this study, a coal mine in Yangquan City, Shanxi Province, China, serves as the research subject to investigate the failure characteristics and evolution of the surrounding rock in an isolated working face. Considering the actual conditions and geological characteristics of the mine, a three-dimensional numerical analysis model was established. This model measures 560 m in length, 320 m in width, and 56 m in height. Displacement constraints were applied to the bottom surface and surrounding interface of the model, along with in-situ stress. A vertical stress of 7.75 MPa was applied to the top of the model, and a lateral pressure coefficient of 1.2 was used. The Mohr–Coulomb criterion was selected as the model's failure criterion. The 15,105 and 15,107 working faces were excavated successively, followed by the excavation of two roadways. Subsequently, the 15,106 working face was mined for 150 m. Figures of the large model and the top view after excavation are shown in Fig. 4. These measures ensure greater accuracy in the simulation results. The rock mechanics parameters and goaf filling parameters used in the simulation are detailed in Table 1 and Table 2, respectively.

**Table 1:** Rock mechanics parameter table.

Parameter Lithology	Density/(kg·m^3^)	Bulk/(GPa)	Shear/(GPa)	Cohesion/(MPa)	Friction/(°)	Tension(MPa)
15 Coal	1300	2.58	1.18	1.23	25	3
Siltstone	2680	13.8	13.6	2.18	30	3.6
Sandy mudstone	2000	8.4	6.8	1.4	30	1.37
Fine-grained sandstone	2410	6.4	2.9	4.1	36	8.1
Shale	1800	4.59	2.3	1.2	13	1

**Table 2:** Goaf filling parameters.

Density/(kg·m^3^)	Bulk/(GPa)	Shear/(GPa)	Friction/(°)	Dilation/(°)
1628	14.37	10.63	2	7

## Results

### Characteristics and evolutionary pattern of roadway damage in different periods of time

The 15,106 working face is an isolated working face. Using the 15,106 transportation roadway as the research subject, we compare the distribution of the plastic failure zone at distances of 0m, 10m, 20m, 50m, 80m, and 100m from the working face during the mining stage with their respective positions during the tunneling stage. This comparison helps in understanding the failure characteristics and spatiotemporal evolution of the surrounding rock of the roadway in this isolated working face. For orientation within the study, the left side of the roadway is designated as the primary side, and the right side as the secondary side.

**Figure 5:**
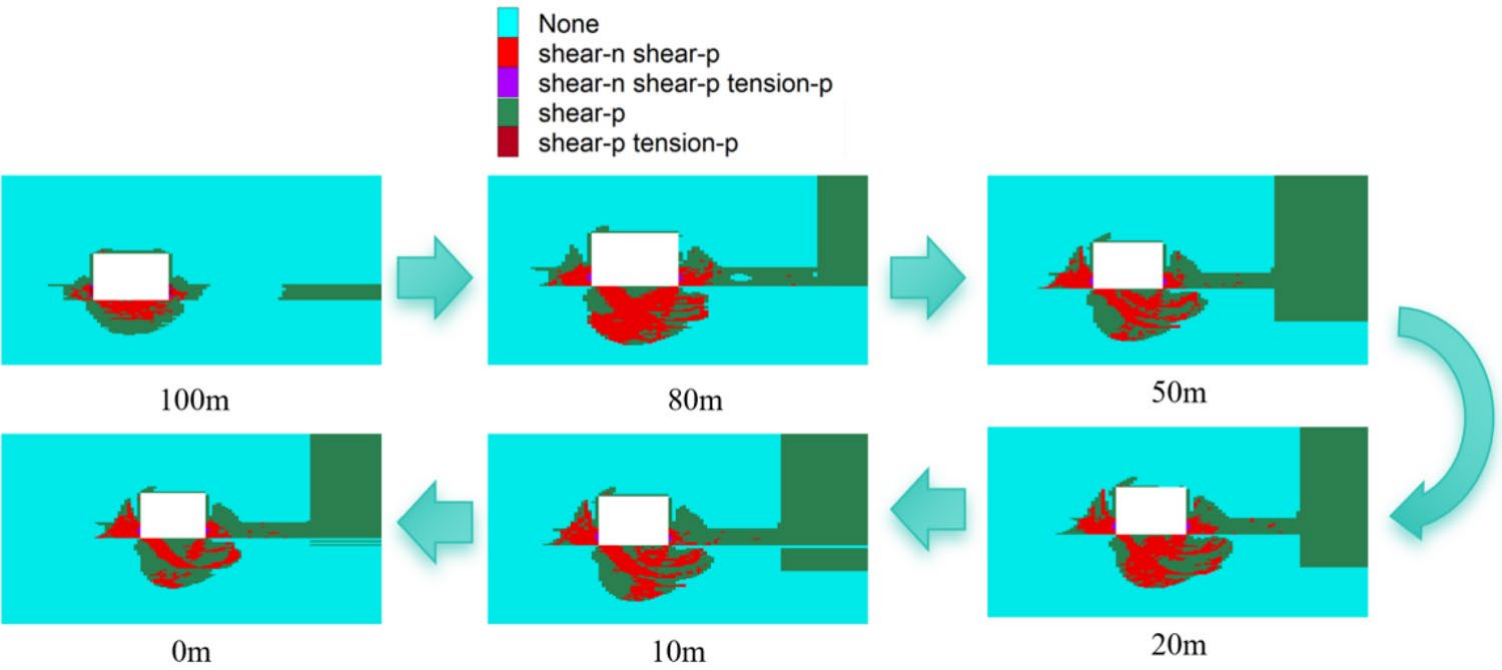
Evolution of the plastic zone of the roadway perimeter rock during the excavation period.

The evolution process of the plastic zone in the roadway's peripheral rock during the excavation period is depicted in Fig. 5. In the 100m to 80m range from the excavation face, the plastic damage area on the roadway roof is small, but the plastic damage areas on both sides of the roadway develop upward and increase in size. The scope and maximum depth of plastic damage are primarily concentrated in the middle-lower positions of both sides, with the maximum depth of plastic damage on the primary side increasing from 2.96m to 3.46m. In comparison, the maximum depth of plastic damage on the secondary side at 100m is smaller, at 2.70m, and the furthest undamaged position from the safety coal pillar is 5.22m. By 80m, the plastic damage area of the secondary side has merged with the safety coal pillar's plastic damage area, forming an "O"-shaped middle section that remains undamaged, with a maximum width of 1.25m. The roadway's floor, composed of a relatively weak coal seam, exhibits extensive plastic damage that tilts toward the right side, resulting in an asymmetric damage pattern. The maximum depth of damage at the bottom plate at 80m is 3.88m, with the nearest undamaged area to the secondary side's damage zone being 0.62m away vertically, and the plastic damage depth of the lower part of the secondary side is 1.88m.

At 0m from the face, there is no noticeable change in the development trend of the plastic damage zone around the roadway. The maximum damage depth of the roof plate is 0.70m. The most severe damage to the gang occurs at the foot, with a maximum depth of 4.24m. The plastic damage zone of the vice gang is already connected with that of the safety coal pillar. The plastic damage area of the roadway's bottom plate extends to the right side, exceeding the position of the vice gang by 3.26m, with a maximum damage depth of 3.92m.

**Figure 6:**
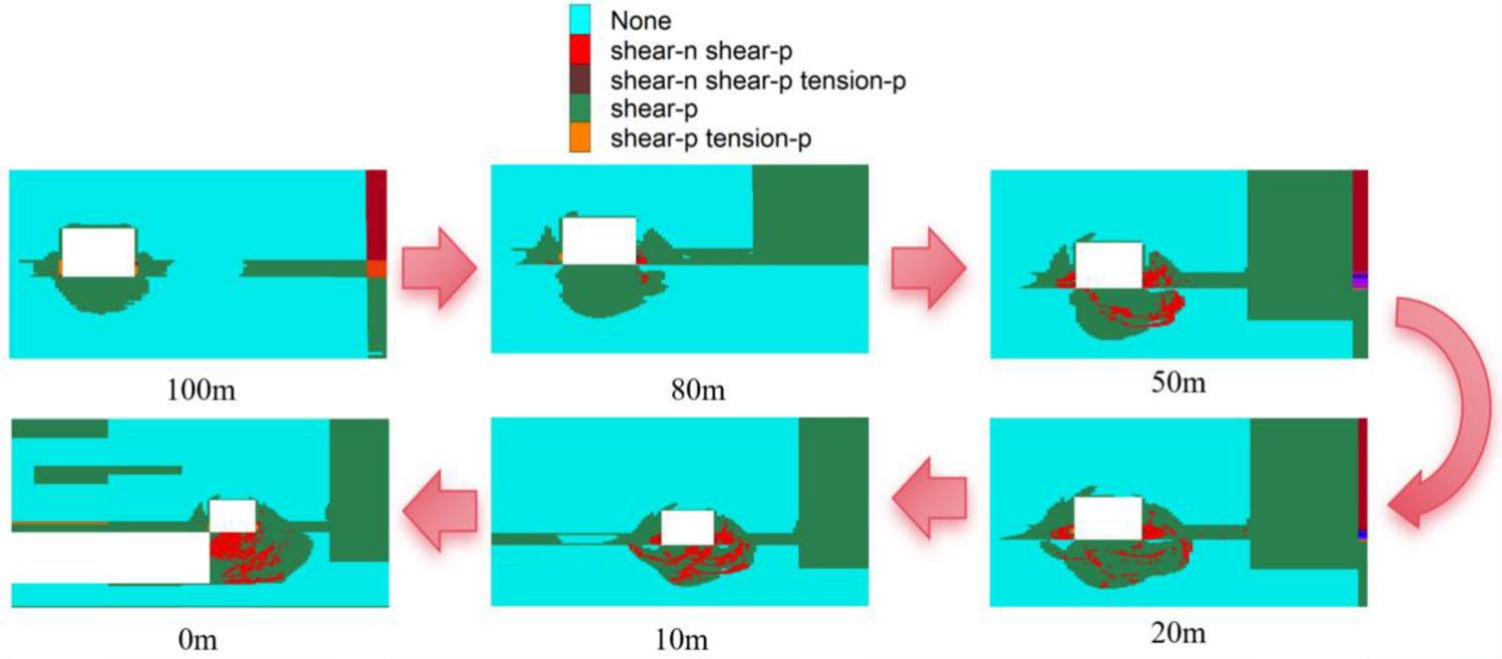
Evolution of the plastic zone of the tunnel perimeter rock during the backhoeing period.

The evolution process of the plastic zone in the peripheral rock of the roadway during the mining back period is shown in Fig. 6. At 100m from the mining face, the range of the plastic damage zone of the roadway roof has not changed significantly. Plastic damage occurs in the middle of the roadway gang. The extent and maximum depth of damage in both gangs are primarily concentrated in the middle and lower sections, influenced by the mining at the face. The maximum depth of plastic damage in the roadway's main gang is 2.99 m. The vice gang, in comparison, has a smaller maximum depth of 2.74 m, and the maximum distance between the safety coal pillar and the plastic damage has been reduced to 5.01 m. The plastic damage area at the bottom of the roadway has further expanded compared to the digging period, and the angle of inclination has also significantly increased, with a maximum depth of damage of 2.68m. As the distance from the working face decreases, the area of plastic damage increases, although the development trend of the plastic damage area of the roof plate and the two gangs does not align with that of the working face. However, the scope and development trend of the plastic damage in the roof and the two gangs have not changed significantly. At 80m, the plastic damage areas of the two gangs have completely merged with the plastic damage area of the safety coal pillar. The maximum damage location in the gangs is in the middle of the lower part of the roadway, with a maximum damage depth of 3.52 m. The bottom plate of the roadway extends to the right side of the plastic damage area, with a maximum perpendicular distance of 0.49 m from the damage area of the two gangs, a maximum depth of plastic damage in the lower part of the roadway of 1.90 m, and a maximum depth of damage to the bottom plate of 3.90 m.

At 50m, there is no significant change in the development trend and scope of the plastic damage area of the roadway's top plate and two gangs. However, the plastic damage area of the roadway has increased, with the maximum damage depth in the main gang reaching 4.24 m. The plastic damage area of the vice gang is now completely connected with the plastic damage area of the safety coal pillar, although it has not yet developed to the top position of the roadway. The plastic damage area of the bottom plate extends 3.26 m beyond the position of the vice gang, with a maximum vertical distance from the vice gang damage area of 0.29 m, and a maximum depth of damage to the bottom plate of 3.91 m.

Within the range of 0 m to 20 m from the mining face, as one approaches the working face, the influence of mining intensifies. On the left side, the roadway roof's plastic damage zone continues to develop towards the upper right, with the maximum depth of damage reaching approximately 0.86m. The plastic damage zones of both gangs of the roadway continue to evolve; the face side of the gang has been completely destroyed, with the most severe damage occurring at the gang's foot, where the maximum depth of damage is 5.47m. The plastic damage zone of the vice gang is now fully connected with the plastic damage zone of the safety coal pillar and has expanded to the top of the roadway. At 10m, the plastic zone of the main gang is nearing connection with the coal damage zone at the front of the workpiece. The plastic damage of the roadway's bottom plate is expanding both leftward and rightward, exceeding the vice gang position by 3.98 m and the face gang position by 3.23 m, with a maximum depth of damage of 4.21 m. At 0m, the maximum depth of damage in the plastic damage area on the left side of the roadway's top plate is 0.91 m, and the plastic damage area of the bottom plate exceeds the vice gang's position by 6.19 m, showing significant destructive depth. The plastic damage area of the bottom plate now exceeds the height of the working face, reaching 5.62 m.

### Surrounding rock fracture development characteristics

**Figure 7:**
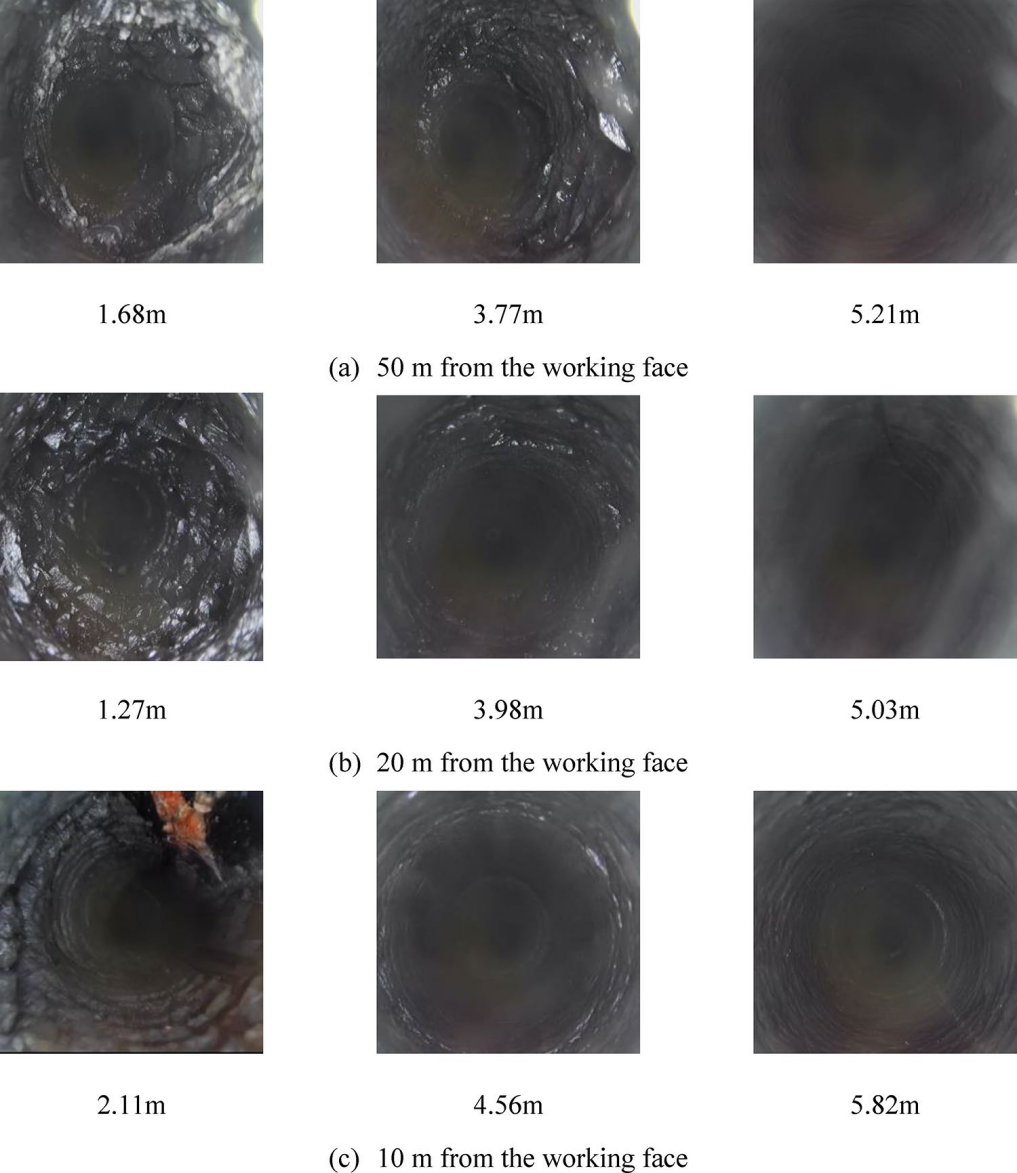
Damage to the surrounding rock at different locations from the working face.

Using a borehole peeper for on-site detection on the main side of the 15,106 transportation roadway, the fracture development and damage within the surrounding rock of the roadway are documented. The damage at different distances from the working face is illustrated in Fig. 7.

As depicted in Fig. 7a, 50m from the working face, the face gang at 1.68 m in the roadway shows severe fragmentation with numerous fissures. At 3.77 m, the coal wall is more extensively broken, featuring a rougher cross-section accompanied by the development of a circular fissure. As probing depth increases, the coal wall at 5.21 m appears more intact, exhibiting only a few micro-fissures.

In Fig. 7b, at 20 m from the working face, the coal wall is seriously fragmented at 1.27 m with many cracks extending deeper. At 3.77 m, the coal wall is further broken, displaying more circumferential cracks, and the entire cross-section is compromised. Deeper at 5.03 m, the coal wall, while not fragmented, shows a longitudinal crack that continues to develop deeper.

As shown in Fig. 7c, 10m from the working face, the face gang of the roadway is fragmented at 2.11 m with both transverse and axial fissures present in larger numbers. There is also a transverse fissure about 0.2 mm wide. At 4.56 m, more circumferential fissures appear around the coal wall, which is rougher overall, and the probing depth reveals continued fragmentation at 5.82 m, where more cracks and a rough coal wall cross-section indicate ongoing damage. At this point, the face gang of the roadway and the coal body damage area in front of the working face appear to be interconnected.

From the results obtained through borehole peeping, the characteristics of the surrounding rock damage development at different distances from the working face were identified. In areas outside the plastic damage zone, the surrounding rock surface is relatively smooth with very few fissures. However, within the developed plastic damage zones of the roadway, the surrounding rock is severely damaged, consistent with the results from numerical simulation calculations.

### Roadway deformation pattern

As the working face advances, the relative displacement of the two sides of the roadway was measured at different locations, providing insights into the pattern of roadway displacement over time. Borehole peeping measurement points were set at 50m, 80m, and 100m from the working face of 15,106. These points correspond to the stages of post-damage development of the plastic zones on both sides of the roadway, penetration damage of the roadway sub-gang, and the stage before penetration damage of the roadway sub-gang, respectively.

**Figure 8:**
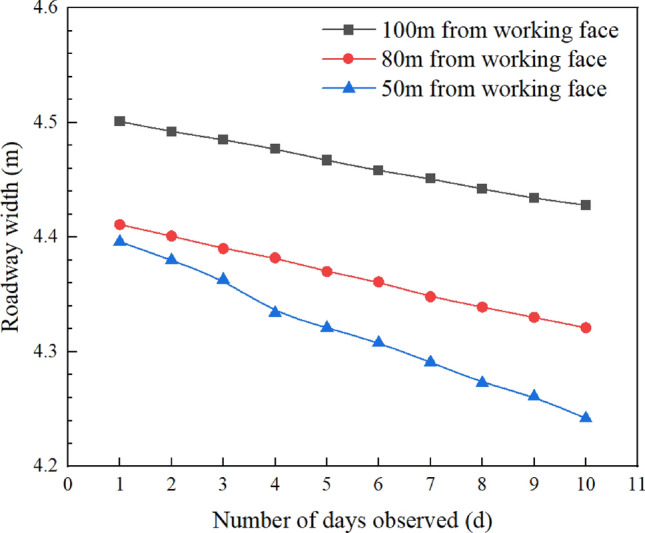
Roadway width over time.

As illustrated in Fig. 8, surface displacement data are plotted in a point-line diagram. Due to the complex geological conditions on site, the measured width of the roadway at these points may not be entirely representative, so only the magnitude of change was analyzed. The roadway width at distances of 50m, 80m, and 100m from the working face decreases over time. The fitted equations are y = − 0.017x + 4.411, y = − 0.010x + 4.421, and y = − 0.008x + 4.509, respectively. The slope of the line, − 0.008x + 4.509, indicates that the absolute value of the slope decreases as the distance from the working face increases, suggesting that the further from the working face, the lesser the impact of mining on the roadway width. At 50 m from the working face, during the post-penetration damage development stage of the plastic zones of both sides of the roadway, the average daily change in roadway width is 17 mm, peaking at 29 mm on the 3rd to 4th days, with a total deformation of 154 mm over 10 days. At 80 m, during the stage of penetration damage of the vice gang of the roadway, the average daily change is 10 mm, peaking at 12mm on the 4th to 5th days, with the rate of change stabilizing thereafter. The total deformation over 10 days is 100 mm. At 100m from the working face, in the stage before penetration damage of the vice gang, the average daily change is 8 mm, peaking at 10 mm on the 4th to 5th days, with a total deformation of 73 mm over 10 days.

Overall, the deformation of the roadway is least in the stage before the sub-gang penetration damage, more pronounced during the penetration damage stage of the sub-gang, and most significant during the post-penetration damage development stage of the plastic zones on both sides of the roadway.

## Discussion

Combining the results from numerical simulations and field tests, it is evident that the size and morphology of the plastic zone along the top of the 15,106 island face vary at different locations on the overpass face during the tunneling and mining periods. In the excavation stage, the distribution of the plastic zone differs at various locations; the closer to the boundary of the peripheral airspace zone, the smaller the size of the plastic zone. Conversely, the farther from the boundary of the peripheral airspace zone, the more asymmetric the expansion of the plastic zone, which eventually connects with the plastic zone of the coal pillar, maintaining a roughly consistent morphology. After mining the 15,106 working face, the evolution of the plastic zone at different locations in the transportation roadway mirrors that of the excavation stage. However, under the influence of mining, the plastic zones are generally larger. The roadway is excavated along the roof with the bottom plate consisting of a softer coal body. At the location 0m from the working face, the depth of the bottom plate destruction reaches 5.62 m. At 10 m from the working face, the plastic zone on one side of the gang begins to connect with the plastic zone of the coal body in front of the working face, continuing until penetration occurs. The roadway's vice gang remains unpenetrated in the initial stage of destruction but is penetrated in the later stage, leading to the development of plastic zones in both gangs of the roadway after the penetration and destruction stage. The asymmetric damage of the roadway exhibits selectivity in the rock layers, characterized by sporadic jumps in damage. The rock layer of the roadway's roof is harder, so the roof has not suffered extensive damage. The on-site test results align with those of the numerical simulation calculations and are consistent with the observed damage in the coal mine.

## Conclusions

In this study, we studied the damage characteristics and evolution of the surrounding rock of the roadway along the top return roadway of the isolated island working face in this mine. The findings are based on on-site measurements using borehole peeping and assessments of the displacement of two roadway gangs, combined with numerical simulation results. The conclusions are as follows:

1. Using numerical simulation, we identified the damage characteristics and evolution of the plastic zone of the roadway along the top of the 15,106 isolated island working face. The evolution of the plastic zone of the roadway of the isolated island working face shows similarities between the excavation and mining periods. Initially, the plastic zones of the two gangs of the roadway expand asymmetrically. At 80 m from the working face, the vice gangs connect with the destruction zone of the coal pillar, while at 10 m from the working face, the main gang gradually connects with the destruction zone of the coal body in front of the working face. The damage depth of the bottom plate is significantly greater than that of the top plate, and the plastic damage area of the gang is predominantly located in the middle and lower parts of the roadway. This aligns with the actual damage observed at the site. The overall experience of the roadway vice gang does not pass through the destruction stage completely; rather, it transitions through the destruction phase, leading to the asymmetric development of the plastic zones in both gangs of the roadway.
2. The closer to the working face, the greater the destruction range and fissure development along the top of the mining roadway of the island working face, leading to overall severe breakage. Within 20 m ahead of the working face, there is fissure development within the inner 5.82 m of the peripheral rock, and the depth of gang destruction is the direct cause of gang bulging.
3. The depth of the plastic damage zone along the top roadway of the island working face is positively correlated with the displacement of the two gangs of the roadway. The size and deformation of the plastic damage zone of the roadway gradually increase with the advancement of the working face. During the on-site monitoring period, the displacement of the roadway continued to increase, with average daily increments at positions of 50 m, 80 m, and 100 m from the working face being 17 mm, 10 mm, and 8 mm, respectively.

## Data Availability

All data and materials are the property of the authors. Corresponding authors may provide raw data upon reasonable request. The datasets used and analysed during the current study available from the corresponding author on reasonable request. You can conducting the study in coal mine.
